# Signal-to-noise trade-offs between magnet diameter and shield-to-coil distance for cylindrical Halbach-based portable MRI systems for neuroimaging

**DOI:** 10.1007/s10334-024-01210-6

**Published:** 2024-10-17

**Authors:** Javad Parsa, Andrew Webb

**Affiliations:** https://ror.org/05xvt9f17 grid.10419.3d 0000 0000 8945 2978 C.J. Gorter MRI Center, Department of Radiology, Leiden University Medical Center, Leiden, The Netherlands

**Keywords:** Ultra Low field MRI, Intrinsic SNR, RF coil, Simulation, Helbach-array MRI magnet

## Abstract

**Objective:**

To investigate the trade-off between magnet bore diameter and the distance between the conductive Faraday shield and RF head coil for low-field point-of-care neuroimaging systems.

**Methods:**

Electromagnetic simulations were performed for three different Faraday shield geometries and two commonly used RF coil designs (spiral and solenoid) to assess the effects of a close-fitting shield on the RF coil's transmit and receive efficiencies. Experimental measurements were performed to confirm the accuracy of the simulations. Parallel simulations were performed to assess the static magnet (${B}_{0}$) field as a function of the magnet bore diameter. The obtainable SNR was then calculated as a function of these two related variables.

**Results:**

Simulations of the RF coil characteristics and ${B}_{1}^{+}$ transmit efficiencies agreed well with corresponding experimentally determined parameters. Overall, the RF coil transmit efficiency was, as expected, higher when the gap between the shield and coil increased. The calculated intrinsic SNR showed that maximum SNR would be obtained for a cylindrical shield of diameter 310 mm with an inner diameter of the magnet of 320 mm (assuming 10 mm for the gradient coils).

**Conclusion:**

This work presents an overview of the trade-offs in transmit efficiencies for RF coils used for POC MRI neuroimaging as a function of coil-to-shield distance and inner diameter of the Halbach magnet. Results show that there is a relatively shallow optimum between a magnet diameter of 290 and 330 mm, with values falling more than 10% if either smaller or larger magnets are used.

**Supplementary Information:**

The online version contains supplementary material available at 10.1007/s10334-024-01210-6.

## Introduction

The number of low-field point-of-care (POC) portable MRI system designs has grown significantly in recent years, with both academic and commercial groups aiming to increase the accessibility of a traditionally extremely expensive imaging modality [1–9] via relatively lightweight, cryogen-free systems with minimal fringe fields and minimal electrical power requirements. The major challenges of portable low-field systems are the low signal-to-noise ratio (SNR) and high ${B}_{0}$ inhomogeneity [10]. A simplified expression for SNR at low fields where coil noise is dominant [11], in terms of ${B}_{0}$ and RF coil receive efficiency is given by:

1 $$SNR\propto {B}_{0}^\frac{7}{4}{B}_{1eff}^{-}$$

where ${B}_{1eff}^{-}$ is the coil receive efficiency, which at low field is essentially identical to the transmit efficiency ${B}_{1eff}^{+}$. In turn, ${B}_{1eff}^{+}={B}_{1}^{+}/\sqrt{R}$, where ${B}_{1}^{+}$ is the ${B}_{1}$ field produced per unit current, and $R$ is the combined resistance of the RF coil and sample.

The transmit/receive efficiencies are maximized by making the RF coil as close fitting to the head as possible. The increased SAR related to such a close-fitting geometry has been shown not to be a problem for POC neuroimaging systems [12]. However, since there is very limited space in neuroimaging POC MRI systems the conductors of the RF coil are intrinsically very close to the inner Faraday shield, required to reduce external electromagnetic interference (EMI) as well as mutual coupling between the RF coil and gradient coils. According to Lenz’s law, mirror currents which are analogous to counter currents located outside the shield, reduce the RF coil transmit/receive efficiency, reducing the SNR with respect to an unshielded coil. The further away the shield from the RF coil the less this reduction, but for a given coil size the further the coil-to-shield distance the larger the inside bore of the magnet which corresponds to a reduction in the ${B}_{0}$ field.

This effect is intrinsic to all geometries of POC systems, irrespective of the frequency at which they are operating. However, the size of the effect does depend on the particular geometry. The largest effect occurs for geometries such as a cylindrical Halbach-based magnet in which a circular or elliptical transmit/receive coil is surrounded by a cylindrical shield on the inside of the gradient coils. In a C-shaped or H-shaped magnet the effect would be less since the gradient coils are typically planar and so any Faraday shield is located further from the transmit coil.

In this work, we consider the case of a Halbach-based system. According to Turek et al. [13] the ${B}_{0}$ field at the center of a dipole Halbach cylinder with finite length $l$, outer radius${r}_{o}$, and inner radius ${r}_{i}$ is given by:

2 $${B}_{0}={B}_{r}\left[\text{ln}\left(\frac{{r}_{o}}{{r}_{i}}\right)-f\right]$$

where ${B}_{r}$ is the remanence of the magnetic material and *f* is defined as:

3 $$f= \text{ln}\frac{l+\sqrt{{l}^{2}+{\left(\frac{{r}_{o}}{{r}_{i}}\right)}^{2}}}{l+\sqrt{{l}^{2}+1}}+\frac{l}{\sqrt{{l}^{2}+{\left(\frac{{r}_{o}}{{r}_{i}}\right)}^{2}}}-\frac{l}{\sqrt{{l}^{2}+1}}$$

To explore the SNR dependence on magnet field strength and transmit efficiency in a neuroimaging Halbach-based system, we present comprehensive electromagnetic (EM) simulations using three commonly employed RF coil setups in combination with analytical simulations of the ${B}_{0}$ field for magnets of different inner diameters. To validate simulation data, we have acquired experimental data on transmit efficiency and image SNR measurements for different shielding setups.

## Material and methods

### Transmit field simulations

**Fig. 1:**
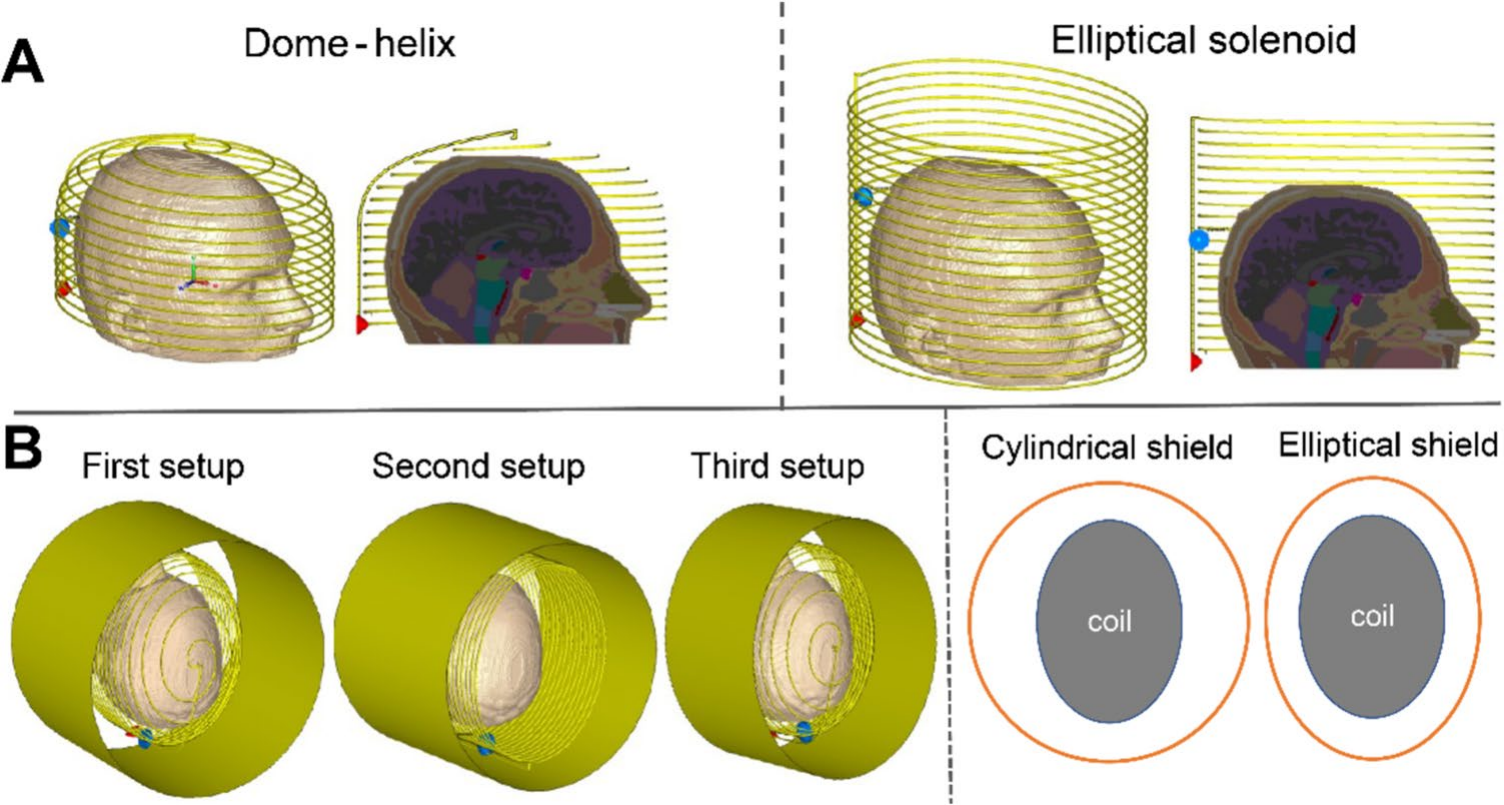
**A** Schematics of the simulated dome-helix (left) and elliptical solenoid (right) coils with the Duke model, from the IT IS Virtual Family. **B** On the left, three different simulation setups are shown, and on the right, a schematic of the coil and the shield (depicted as an orange circle) is presented

EM simulations were performed in CST Microwave Studio (CST GmbH, Darmstadt, Germany). Two different RF coil geometries were simulated, each operating in transmit/receive mode. The first is a dome-helix [14] and the second is an elliptical solenoid coil, consisting of 15 and 20 turns of copper wire of 1.5 mm diameter, respectively, with one capacitive segmentation halfway along the wire length. The coils had dimensions of 180 mm width, 240 mm height, and 10 mm gap between wire turns (Fig. 1A). The coils were loaded with the head model, Duke, from the IT IS Virtual Family [29], with an isotropic resolution of 1 mm × 1 mm × 1 mm. Its material properties were categorized into 18 different tissues to simplify the number of dielectric properties in the model; for example, the eye, sclera, cornea, and vitreous were considered to have the same conductivity/permittivity/proton density. Two different geometries, cylindrical and elliptical, of the RF shield were simulated as a continuous copper structure with a length of 350 mm, a thickness of 0.07 mm, and a conductivity of $5.96\times {10}^{-7} S/m$. The time domain solver with open boundary conditions in all directions was used, 1-W input power was considered for all simulations, and the computations were ended at an accuracy of − 40 dB. Figure 1B shows the three different simulation setups considered:

- First setup: dome-helix coil inside a cylindrical shield with various diameters from 260 to 320 mm with a 5 mm step size.
- Second setup: an elliptical solenoid coil inside a cylindrical shield with various diameters from 260 to 320 mm with a 5 mm step size.
- Third setup: a dome-helix coil inside an elliptical shield with a symmetric gap between the coil and shield where the major axis changes from 260 to 320 mm with a 5 mm step size.

In each case, the RF coils were impedance matched to 50 Ω at 1.965 MHz using variable capacitors in an L-network. The ${B}_{1}^{+}$ efficiency ($\mu T/\sqrt{W}$) map was calculated for each setup.

#### Experimental measurements 

**Fig. 2:**
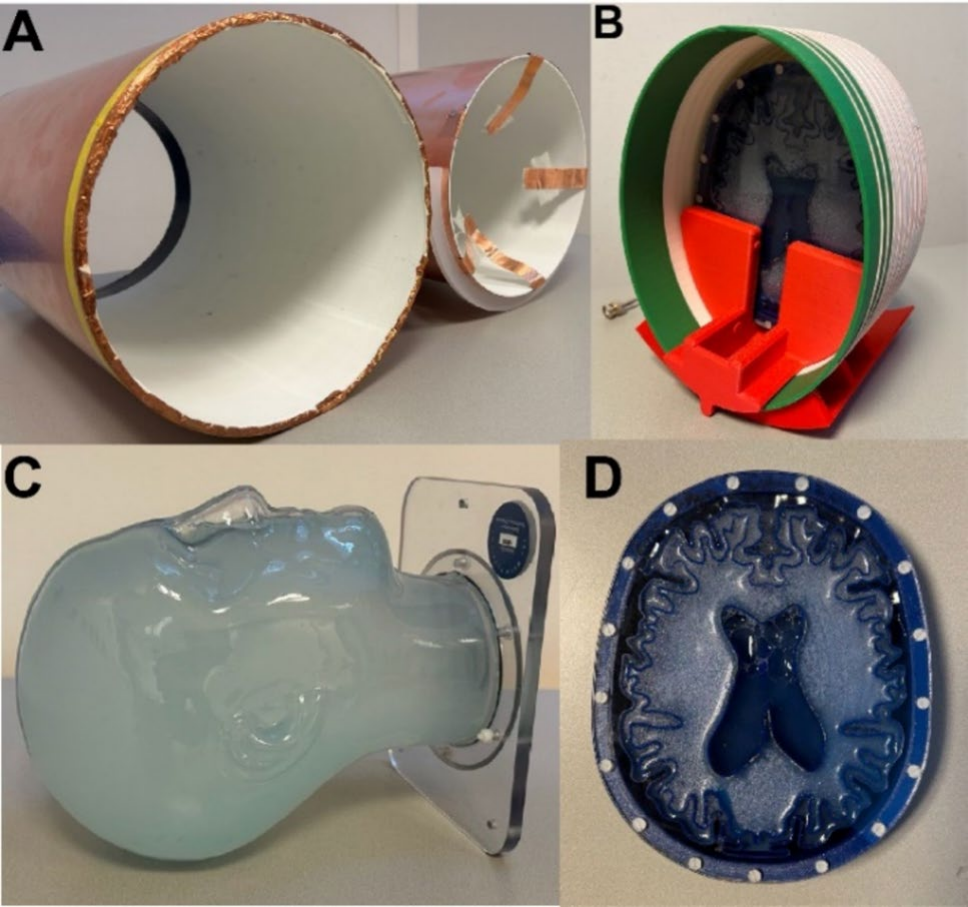
An illustration of the imaging setups for the TSE and B_1^ + maps on the Halbach POC low field system. **A** two shields 300 mm and 260 mm in diameter. **B** Constructed coil with slab phantom inside. **C** Head model phantom filled with copper sulphate doped water (T1 ≈ 100 ms) used for B_1^ + mapping. **D** Two-dimensional brain phantom corresponding to axial brain slabs with a thickness of 20 mm and relaxation parameters of the compartments approximately equal to in vivo data at 50 mT [17]

To validate the simulated data, two turbo spin echo (TSE) images and ${B}_{1}^{+}$ maps were acquired on a Halbach POC low field system [6, 15] using a dome-helix coil placed within a cylindrical shield of different diameters, 300 mm and 260 mm, each 0.07 mm thick, (Fig. 2). ${B}_{1}^{+}$ maps were acquired from a head model phantom filled with copper sulphate doped water (T_1_ ≈ 100 ms) the phantom dimension is 240 mm in the head/foot direction, 190 mm anterior/posterior, and 160 mm left/right. ${B}_{1}^{+}$ mapping used a 3D double angle (60° and 120°) method (DAM) [16], 600 ms/8 ms repetition time/echo time (TR/TE), field-of-view of 220 × 210 × 210 mm^3^, spatial resolution 4 × 4 × 4 mm^3^, 20 kHz acquisition bandwidth, and a 200 microsecond RF pulse duration. K-space data were filtered using a sine-bell function. ${B}_{1}^{+}$ maps were produced from the tip angle (α) maps:

4 $$\alpha = {\text{cos}}^{-1}\left(\frac{{s}_{2}}{2{s}_{1}}\right)=\tau \gamma {B}_{1}^{+}\stackrel{{\phantom{a}}}{\Rightarrow } {B}_{1}^{+}=\frac{1}{\tau \gamma }{\text{cos}}^{-1}\left(\frac{{s}_{2}}{2{s}_{1}}\right)$$

where ${S}_{1}$ and ${S}_{2}$ represents the signal intensities from the 60° and 120° image acquisitions, τ indicates the RF pulse duration and $\gamma$ is the gyromagnetic ratio. Background noise outside the phantom was masked.

To validate simulated SNR results, two turbo spin echo (TSE) images from a brain slab phantom (Fig. 2) with dimensions of 200 × 150  × 20 mm were obtained. 2D TSE images were acquired with 1000 ms/20 ms TR/TE, field-of-view 220 × 200 mm^2^, spatial resolution 1 × 1 mm^2^, 20 kHz acquisition bandwidth, 10 averages, and a 200 microsecond RF pulse duration.

### Static magnetic field simulations

Magnetostatic simulations were conducted using Python, building upon the framework established by O’Reilly [18], to obtain the magnetic field strength and homogeneity within a 200 mm diameter spherical volume (DSV). The magnet is based on an existing Halbach-based neuroimaging POC system with a length of 496 mm [15]. Based on the range of shield diameters simulated from 260 to 320 mm, and assuming a 10 mm thickness to account for the presence of the three gradient coils, the magnet inner diameter was changed in eight steps from the smallest possible 270–330 mm. Within the Halbach array, each ring comprised 12 × 12 × 12 ${\text{mm}}^{3}$ N48 neodymium boron iron (NdBFe) magnets, arranged in a dipolar (k = 2) Halbach configuration. In this design each ring contains two layers of magnets and the outer layer has seven more magnets than the inner layer. In all cases, the outer diameter is 81 mm larger than the inner diameter and the number of cubic magnets increases as the diameter of each ring is increased. Finally, by combining the transmit efficiency and magnetic field strength, the SNR of the coil was calculated.

## Results

### Transmit field simulations and experimental data

${S}_{11}$ Reflection coefficients of three simulation setups along with a comparison with measured ${S}_{11}$ data are shown in Supplementary Fig. 1. There is a good agreement between simulated and measured ${S}_{11}$ in the Dome-helix coil placed inside 300 mm and 260 mm cylindrical shield setups with an overall error of ~ 2.5% in the frequency bandwidth. The measured loaded-to-unloaded Q ratio for the dome coil inside the 300 mm and 260 mm cylindrical shield is 0.69 and 0.61, respectively.

**Fig. 3:**
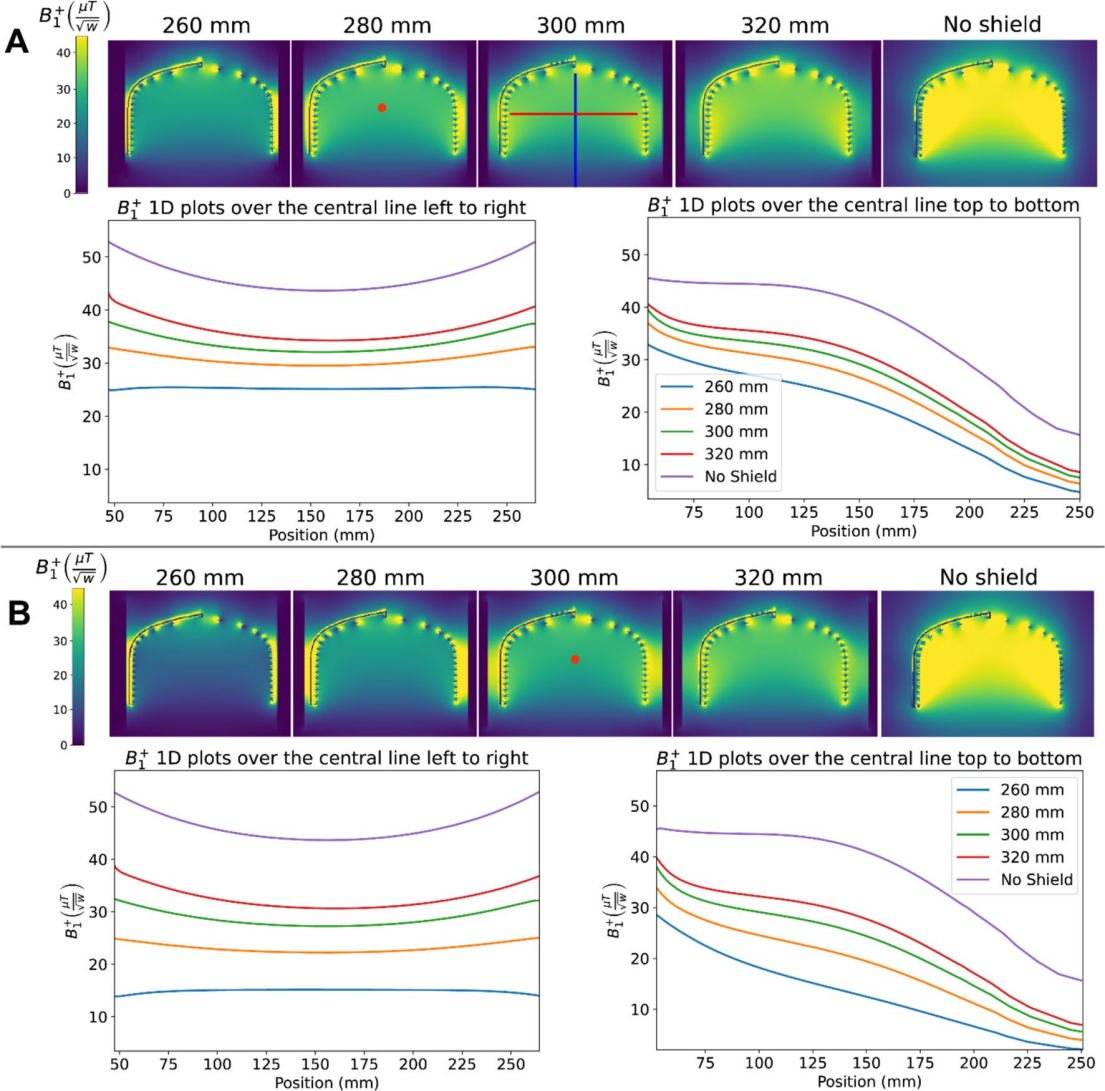
Sagittal slice of simulated B_1^ + efficiency maps as a function of shield diameter for **A** First setup: the dome coil inside a cylindrical shield, and **B** Third setup: the dome coil inside an elliptical shield. The red dot represents the center of the coil

**Fig. 4:**
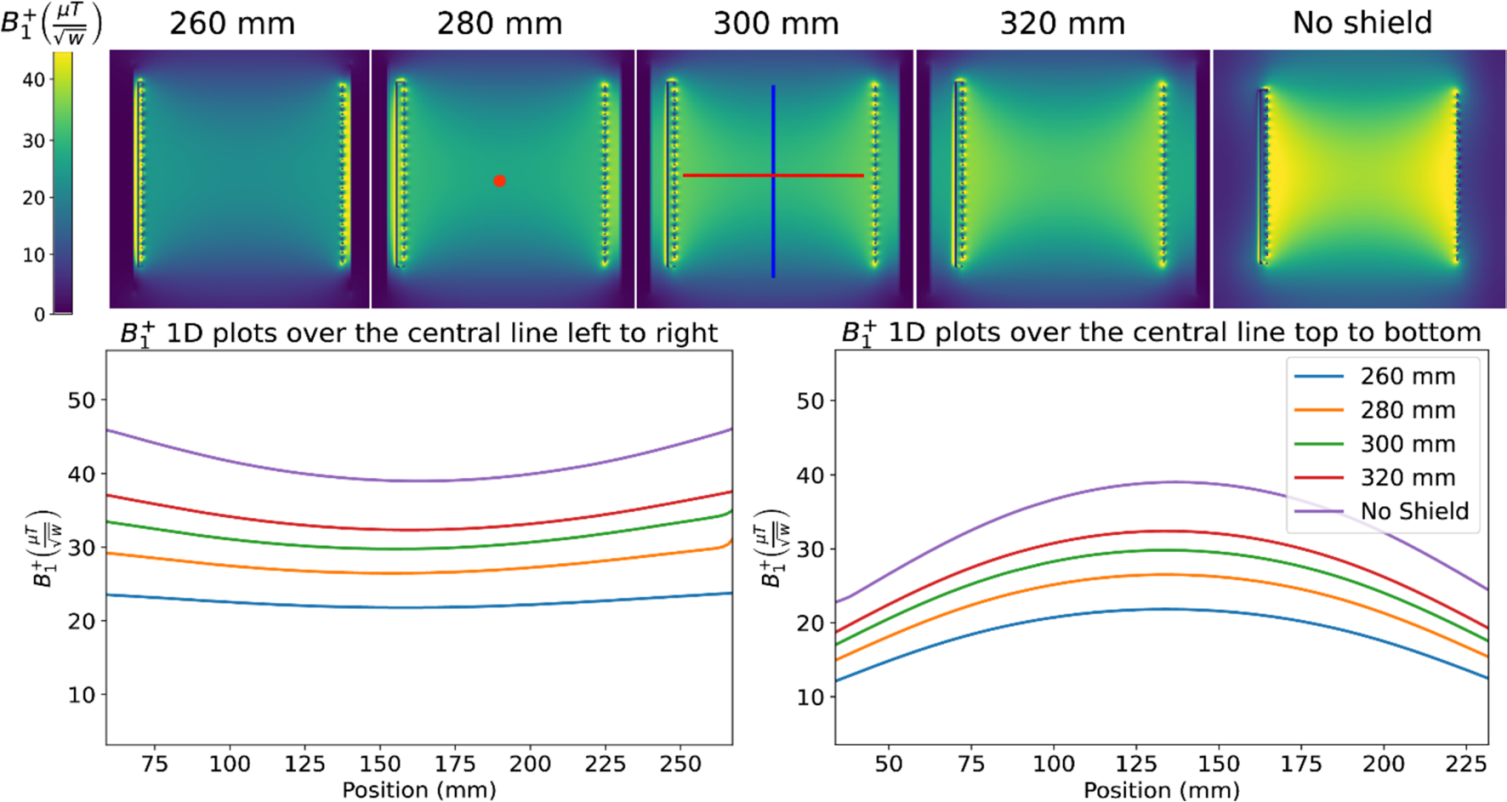
Sagittal slice of simulated B_1^ + efficiency maps as a function of shield diameter for the second setup, featuring the elliptical coil within a cylindrical shield of varying diameters. The red dot represents the center of the coil

Figures 3 and 4 show the simulated ${B}_{1}^{+}$ efficiencies for the three different shielding setups. For the dome helix with a cylindrical shield, the ${B}_{1}^{+}$ efficiency at the center of the coil (the red dot in Figs. 3 and 4) drops from 34.2 to 25.1 $\mu T/\sqrt{W}$ for a 320 and 260 mm diameter shield, respectively, corresponding to a ~ 27% reduction in SNR at that point. The same coil with an elliptical shield shows significantly poorer performance with transmission efficiency at the center point drops from 30.6 to 15.1 $\mu T/\sqrt{W}$. Results for the elliptical solenoid with cylindrical shield are similar to the dome-helix with efficiency dropping from ~ 32.6 to ~ 21.8 $\mu T/\sqrt{W}$, but with a less pronounced drop-off in sensitivity in the head-feet direction due to the longer length and higher axial symmetry.

**Fig. 5:**
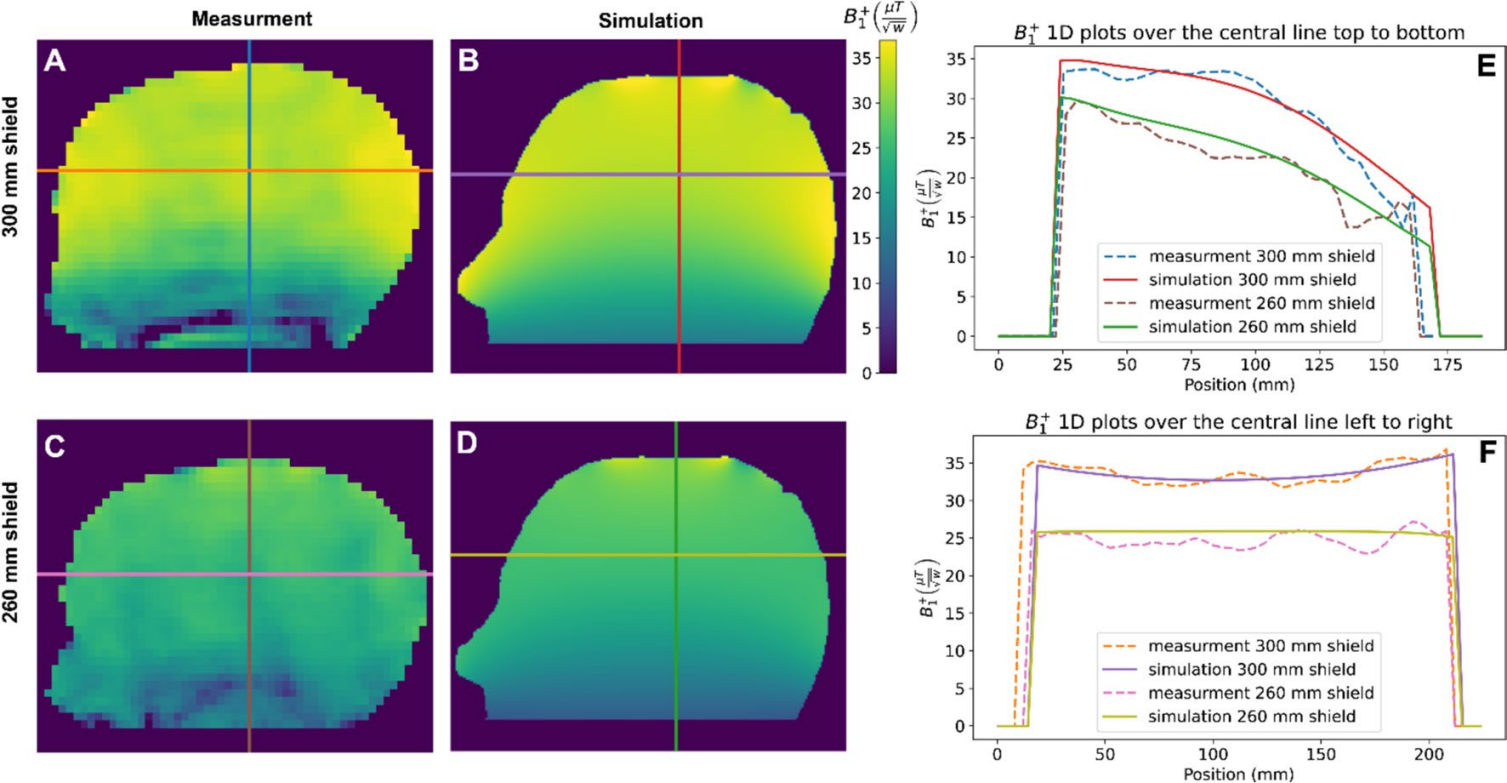
A comparison between simulation and measurement data for the dome helix coil inside the cylindrical shield (first setup). **A**, **B**, **C**, **D** Central sagittal plane of the B_1^ + map of the coil from simulation (**B**, **D**) and measurement (**A**, **C**) data for two different cylindrical shield sizes condition, 300 mm (**A**, **B**) and 260 mm(**C**, **D**) diameter. **E**, **F** Corresponding 1D plot for lines from top to bottom (**E**) and right to the left (**F**). Note: B_1^ + efficiency map was masked to remove the background noise

Figure 5 compares the measured and simulated ${B}_{1}^{+}$ efficiency map in three central slices for the dome coil located inside a cylindrical shield of two different diameters, 300 mm, and 260 mm. The one-dimensional projection plots show reasonable agreement between the experimental and simulation data, with a slightly sharper drop-off in the head-foot direction seen in the experimental data. The mean values of the ${B}_{1}^{+}$ efficiencies over the sagittal slices are 30.2 $\mu T/\sqrt{W}$ (300 mm shield) and 23.4 $\mu T/\sqrt{W}$ (260 mm shield) for measurement data, and 29.5 $\mu T/\sqrt{W}$ and 22.4 $\mu T/\sqrt{W}$ for corresponding simulation data.

**Fig. 6:**
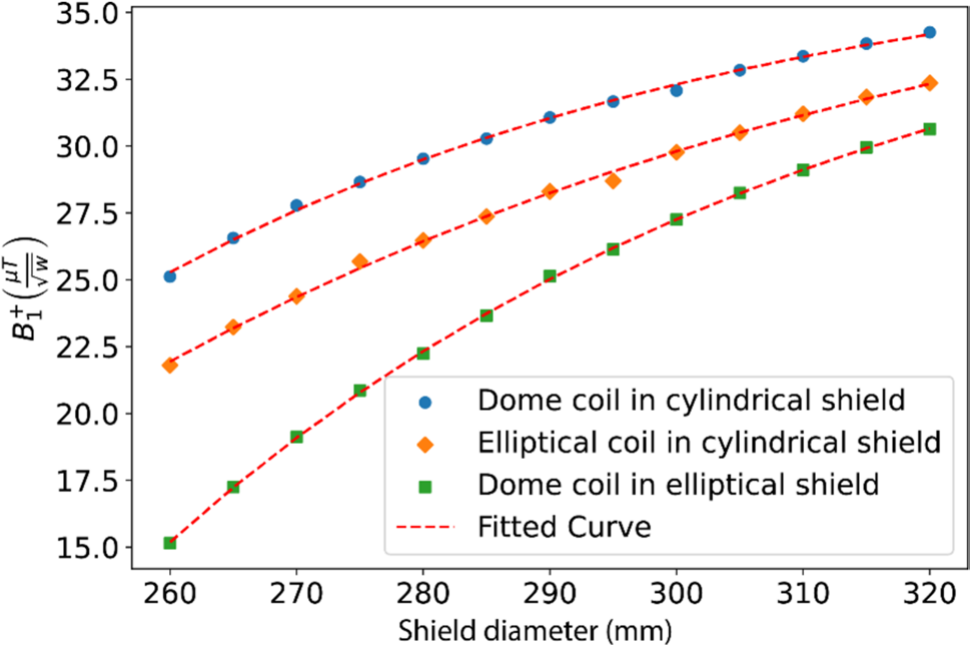
Simulated B_1^ + efficiency at the central point of the coil (represented by red dots in Figs. 3 and 4) for different shield sizes and shapes for all three setups

Figure 6 plots the simulated ${B}_{1}^{+}$ efficiency at the center of the coil represented by a red dot in Figs. 3 and 4. The results show that there is a stronger drop-off in transmit efficiency for the dome coil inside an elliptical shield compared to a cylindrical shield. The results show that the relationship between efficiency and diameter can be well approximated by simple exponentials.

**Fig. 7:**
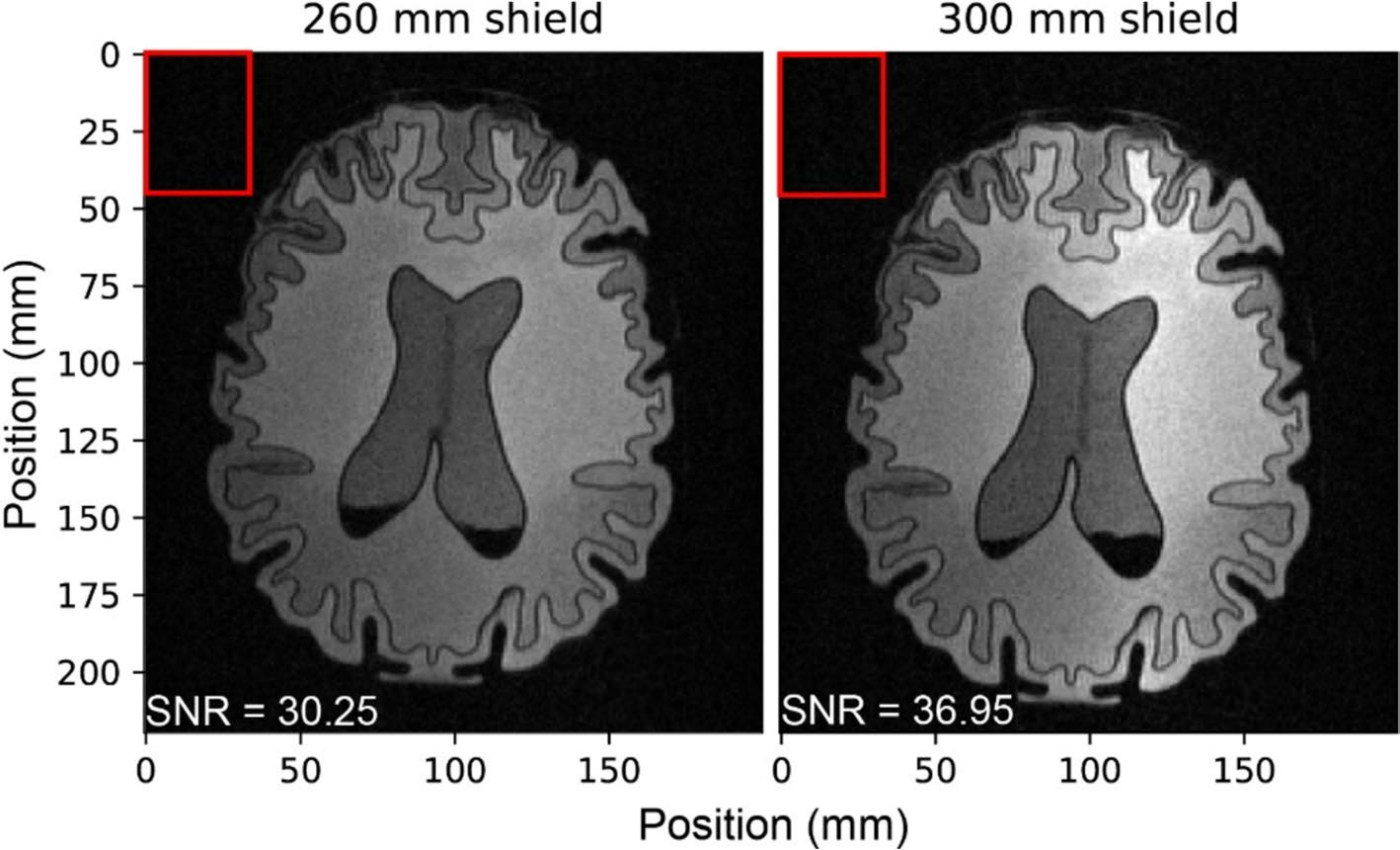
An experimental comparison between 2D TSE images from brain slab in 300 mm and 260 mm shield diameter. The red rectangle represents the STD of the background noise and black region in the center of the image is air

Figure 7 presents an experimental comparison of 2D TSE images from a brain slab acquired using 300 mm and 260 mm shield bores using the dome-helix coil inside the cylindrical shield. The SNR in the images was measured by dividing the mean value of the signal in the imaging area by the standard deviation of the background noise. The SNR of the brain slab image decreased by 18.67%, from 36.95 in the 300 mm cylindrical shield to 30.05 in the 260 mm shield. This reduction corresponds with the results shown in Fig. 6, where the transmit efficiency of the dome coil inside the 300 mm and 260 mm cylindrical shields was 32.01 $\mu T/\sqrt{W}$ and 25.11 $\mu T/\sqrt{W}$, respectively. Under constant magnet strength conditions, this translates to a 21.55% reduction in SNR.

### Static magnetic field simulations

**Fig. 8:**
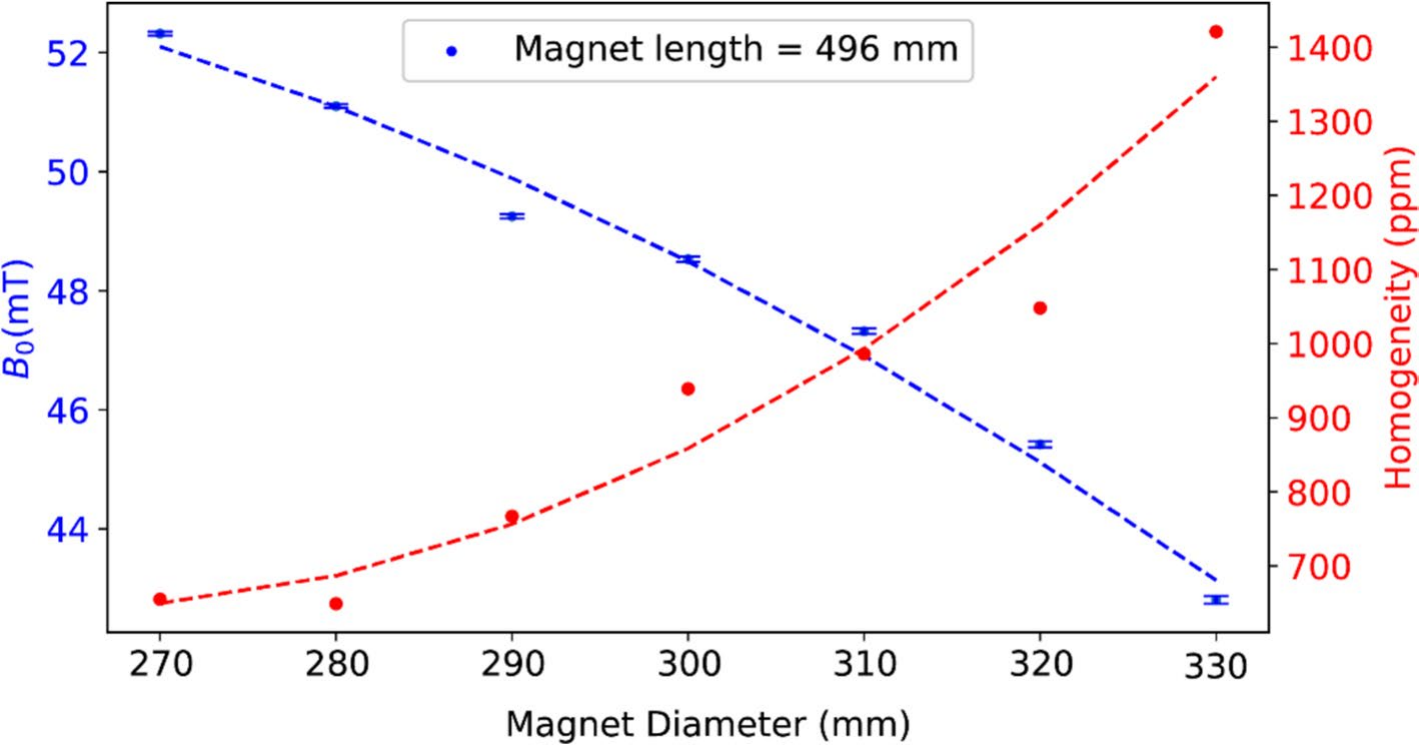
Simulated analytical calculation of magnetic field with Python-based method introduced by O’Reilly [18]. Changes in average magnetic field strength (blue dots) and homogeneity (red dots) with respect to the magnet diameter—the inner diameter of the magnet is 10 mm larger than the shield diameter

As mentioned in the introduction, the coil-to-shield distance can be increased by increasing the inner diameter of the magnet. Figure 8 displays the average magnetic field strength (${B}_{0}$) and homogeneity across within a 200 mm DSV for different magnet inner diameters. The magnetic field strength in the DSV fell by 18.5%, from 52.3 mT to 42.7 mT and inhomogeneity increased by 115% from 665 to 1421 ppm, with an increase in diameter from 270 to 330 mm.

**Fig. 9:**
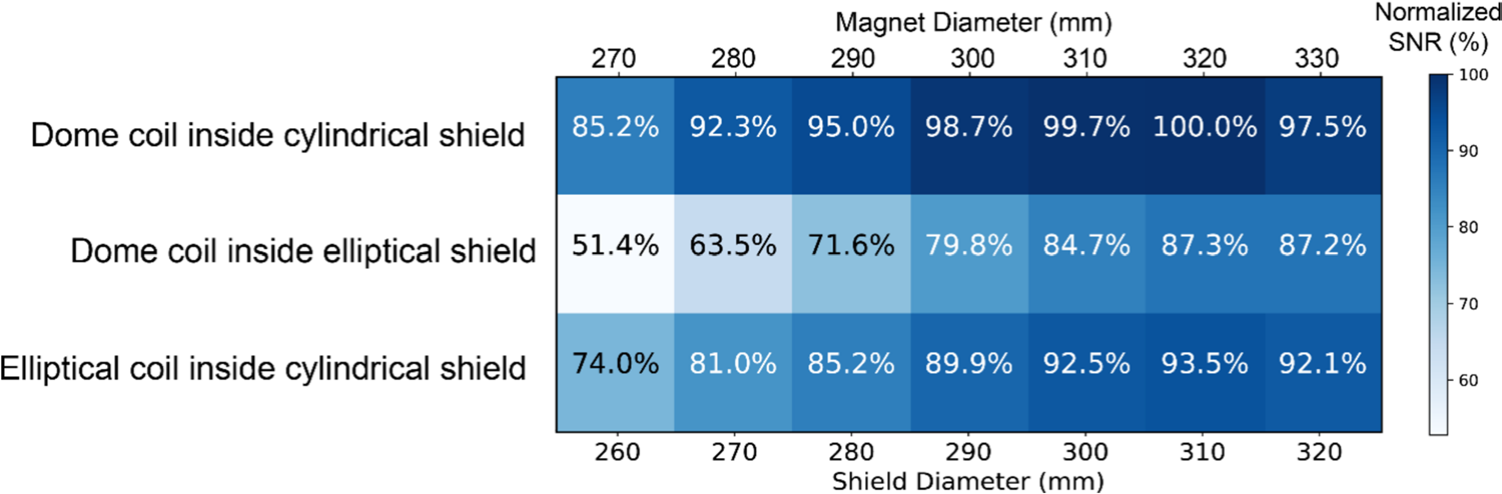
Normalized intrinsic SNR comparison between seven different magnet and shield sizes for all simulation setups

Combining the simulation results from the transmit efficiency and shield/magnet diameter according to Eq. (1), Fig. 9 shows that the maximum SNR would be obtained by using a dome coil inside a cylindrical shield of diameter 310 mm, with a corresponding magnet inner diameter of 320 mm. Although reducing the magnet diameter to 270 mm would increase the signal by more than 18% due to the effect of ${B}_{0}$ alone, the associated reduction in transmit/receive efficiency means that there is an overall reduction in SNR of over 12%.

## Discussion

This work has investigated via simulations and experiments the effects of shield-to-coil distance on the SNR in Halbach-based POC MRI systems for neuroimaging by considering the effects of RF coil transmit efficiency and the attainable ${B}_{0}$ field. Whereas one might expect that the dominant term in determining SNR is the ${B}_{0}$ field, and that therefore the SNR would be maximized by using the smallest possible magnet diameter, our results show that if the magnet is designed to fit very snugly around the patient, then the correspondingly small shield-to-coil distance reduces the transmit/receive sensitivity to an extent that over-rides the ${B}_{0}$-related SNR improvement. Therefore, it is important to calculate both effects and to derive an optimum value of the two related parameters. The results in Fig. 9 show that the normalized SNR close to the optimum value is a relatively shallow function, and so one can choose a magnet diameter towards the lower end if weight/cost are the major concerns, or towards the upper end if patient comfort and space are more important. These general results are not specific to low field strengths, since the transmit efficiency is reduced irrespective of whether coil or sample noise is dominant. As outlined in the introduction, the largest effect occurs for cylindrical Halbach-based magnets, whereas in a C-shaped or H-shaped magnet the effect would be less, since the shield is further away from the transmit coil. In addition to changes in the ${B}_{1}$ field, of course, there are also changes to the electric field distribution, although these are not as important as at higher fields due to their very low magnitudes: these are shown in Supplementary Fig. 3.

There are many possible coil geometries for POC systems, including cylindrical and elliptical. A simulated comparison between elliptical and cylindrical solenoids indicates a 75% higher transmit efficiency for the elliptical solenoid, as shown in Supplementary Fig. 4, due to the larger overall gap between the coil and the shield. One can also introduce non-uniform spacing between coil windings, with tighter winding at the end of the coil, to improve B1 uniformity [19]. In this case, the effects of shield diameter on transmit efficiency should be essentially identical to the uniformly wound cases considered here. The data presented show that, as expected, a cylindrical shield produces less reduction in transmit efficiency than an elliptical shield for elliptical RF coil geometries since the latter has one axis closer to the RF coil and thus induces higher mirror currents. In this work, we did not investigate the design of elliptical magnets, although this might be one configuration in which an elliptical shield geometry would make sense[20]. Our analyses also considered a magnet with a fixed length (496 mm) and variable diameter, as opposed to a series of magnets with a fixed length-to-diameter ratio. This means that there is potentially an additional factor that might affect SNR, namely the degree of ${B}_{0}$ inhomogeneity, necessitating more ${B}_{0}$ shimming. The distribution of ${B}_{0}$ across seven different magnet sizes in a 20 cm^3^ DSV, including three central slices, is shown in Supplementary Fig. 2: the homogeneity of each magnet is shown in Fig. 8.

In this study, we modeled the RF shield as a solid conducting structure, but prior research, including references [21–23], has explored alternative shielding structures such as active shielding. This approach uses wire winding patterns that generate opposing surface current distributions, effectively maintaining a uniform ${B}_{1}$ field. Despite some advantages, this method still reduce the transmit efficiency of the coil and, in addition, active shielding is less suitable for portable MRI systems due to increased heat dissipation and added complexity. An alternative passive method involves frequency-selective surfaces (FSS) with capacitive mesh grids, as demonstrated by Rajendran et al. [24], using a cylindrical high-pass FSS shield around a small solenoid to filter out RF frequencies below 1.5 GHz in a 67 mT Halbach-based POC system. Although this technique boosts transmission efficiency, it falls short of the performance of a continuous solid copper sheet in terms of blocking external noise.

Other factors such as magnet size and homogeneity, and gradient strength also affect the image quality. One feature of POC systems is that they are ideally portable and so the magnet is very small compared to many of the very low field systems introduced in the 1980s which produced diagnostic quality images from field strengths as low as 20 mT [25, 26]. In these cases, the RF shield was a significant distance from the RF coil, and so essentially full ${B}_{1}$ transmit efficiency was possible. However, these systems were generally not portable, with very heavy magnets. In POC systems there is a much closer relationship between the magnet, gradient and RF coil dimensions, since these all fit very tightly within one another, and so the co-dependencies of performance are much more tightly coupled. de Vos et al.[27] have presented a much more sophisticated iterative approach where the entire system can be designed given specific imaging performance requirements. We note that this paper, in fact, did not consider the shield-to-coil distance in terms of calculating transmit efficiency, and so ideally the results from this paper should be incorporated into the approach of de Vos et al.

## Supplementary Information

Below is the link to the electronic supplementary material.

Supplementary file1 Supplementary Figure 1. Simulated S11 three setups with different shield diameters showing reflection coefficients around −30 dB. A) Comparison between simulated S11 and measured S11 for Dome coil inside a cylindrical shield for two different shield sizes. B) Simulated S11 of Dome-helix coil inside the cylindrical shield with different shield sizes (fist setup). C) Simulated S11 of elliptical solenoid coil inside the cylindrical shield (second setup). D) Simulated S11 of Dome-helix coil inside the elliptical shield with different gaps between shield and coil (third setup). Note: all the Bandwidths (BW) are in kHz. (EPS 2688 KB)

Supplementary file2 Supplementary Figure 2. Simulated axial, transverse, and longitudinal central slices within a 200 mm DSV, showing the B0 field with respect to the value at the center from magnet arrays with different inner diameters as indicated on left, outer diameter for each magnet is 81 mm larger than the inner diameter. From top-to-bottom, the B_0 value at the center is 52.32 mT, 51.1 mT, 49.25 mT, 48.53 mT, 47.32 mT, 45.42 mT, and 42.81 mT, respectively. Homogeneity from top to bottom is 655, 649, 767, 939, 986, 1048, and 1421, respectively. (EPS 2152 KB)

Supplementary file3 Supplementary Figure 3. Simulated electric field changes inside the head with respect to the shield diameter for the three setups. Electric filed mean value inside head model is written for every setup and shield diameter in the figure. (EPS 3059 KB)

Supplementary file4 Supplementary Figure 4. A simulation comparison between the transmit efficiency of a cylindrical and an elliptical solenoid inside a 300 mm diameter shield. Central sagittal (A) and axial (C) planes of the simulated B_1^+ map of two coils. B and D) Corresponding 1D plot for lines from top to bottom (B) and right to the left (C). These results show using the elliptical coil has 75% higher transmit efficiency at the center of the coil, ~30 μT⁄√W vs ~17 μT⁄√W. (EPS 2759 KB)

## Data Availability

All data and code that support the findings of this study are available from the author, upon request.
